# Complete Genome Sequence of a Street Rabies Virus Isolated from a Dog in Nigeria

**DOI:** 10.1128/genomeA.00214-12

**Published:** 2013-02-21

**Authors:** Ming Zhou, Zutao Zhou, Grace S. N. Kia, Clement W. Gnanadurai, Christina M. Leyson, Jarlath U. Umoh, Jacob P. Kwaga, Haruna M. Kazeem, Zhen F. Fu

**Affiliations:** a Department of Pathology, College of Veterinary Medicine, University of Georgia, Athens, Georgia, USA; b State Key Laboratory of Agricultural Microbiology, College of Veterinary Medicine, Huazhong Agricultural University, Wuhan, China; c Department of Veterinary Public Health and Preventive Medicine, Ahmadu Bello University, Zaria, Nigeria; d Department of Veterinary Microbiology, Faculty of Veterinary Medicine, Ahmadu Bello University, Zaria, Nigeria

## Abstract

A canine rabies virus (RABV) was isolated from a trade dog in Nigeria. Its entire genome was sequenced and found to be closely related to canine RABVs circulating in Africa. Sequence comparison indicates that the virus is closely related to the Africa 2 RABV lineage. The virus is now termed DRV-NG11.

## GENOME ANNOUNCEMENT

Rabies, which can cause an almost invariably fatal encephalomyelitis, remains a public health threat around the world. Although effective vaccines are available, more than 55,000 people still die from rabies each year throughout the world, with most of them in developing countries of Asia and Africa (1). Rabies virus (RABV) belongs to the genus *Lyssavirus* in the *Rhabdoviridae* family. Its genome is a single-stranded negative-sense RNA of approximately 12 kb in length (2, 3). In previous studies, on the basis of phylogenetic analysis of the nucleoprotein (N) and/or the glycoprotein (G) gene sequences, all RABVs (genotype 1 of lyssaviruses) have been divided into two major clades, one comprising those isolated from terrestrial animals around the world and the other containing viruses isolated from bats and raccoons in the Americas (4–7). A strain of RABV was isolated from a dog brain originating in Nigeria and was designated DRV-NG11. To obtain the whole genomic sequence of this virus, total RNA was extracted from dog brain using TRIzol LS reagent (Invitrogen, Carlsbad, CA). Ten pairs of oligonucleotide primers for amplifying regions of the RABV genomes were designed as previously described (8–11). All PCR products were purified using a QIAquick gel extraction kit (Qiagen, Germantown, MD) and then cloned into the pCR-Blunt II vector (Invitrogen). Cloned DNA was sequenced using a BigDye Terminator cycle sequencing ready reaction kit and an ABI Prism 3730 sequencer. The genomic sequence was assembled by using SeqMan software (DNASTAR Inc.). Homology searches and comparisons of all the sequences obtained were carried out with the aid of the Lasergene package (DNASTAR Inc.). Sequences of encoded proteins were aligned using MEGA version 5 (12). The complete genome of DRV-NG11 is 11,923 nucleotides (nt) in length, similar to genomes of other street RABVs published to date (10, 11, 13). The lengths of the coding sequences are as follows: 1,353 nt for the nucleoprotein (N), 894 nt for the phosphoprotein (P), 609 nt for the matrix protein (M), 1,575 nt for the glycoprotein (G), and 6,384 nt for the RNA-dependent RNA polymerase (L) genes. Comparison of N gene sequences indicates that this virus is related to another RABV identified in Nigeria (EU038098) with high homology (99.4%) and thus it belongs to the Africa 2 lineage (14, 15). The homology between this RABV and those in the “cosmopolitan” group ranges from 86.5% to 87.8% (14, 15). To our knowledge, this is the first report of a complete genome sequence of RABV isolated from Nigeria. The complete genome analysis of DRV-NG11 will advance further studies in rabies epidemiology and pathogenesis.

### Nucleotide sequence accession number. 

The complete genome sequence of DRV-NG11 is available in GenBank under accession number KC196743.
